# Supplementary material for: The Systems Biology Research Tool: evolvable open-source software
Source: BMC Syst Biol. 2008 Jun 29;2:55. doi: 10.1186/1752-0509-2-55 (PMC2446383; doi:10.1186/1752-0509-2-55)
Supplement: Additional file 1 — SBRT Archive. An archive of the current version of the Systems Biology Research Tool. [file 1752-0509-2-55-S1.zip › sbrt-1.4.0/doc/developers_guide/api/sbrt/kernel/package-use.html]

Uses of Package sbrt.kernel


|  |  |  |
| --- | --- | --- |
| **Overview** \| **Package** \| Class \| **Use** \| **Tree** \| **Deprecated** \| **Index** \| **Help** | |  |
| PREV   NEXT | **FRAMES**    **NO FRAMES**     **All Classes** |  |


---


## **Uses of Package sbrt.kernel**

| Packages that use sbrt.kernel | |
| --- | --- |
| **sbrt.kernel** | Provides interfaces to define processes. |
| **sbrt.kernel.fba.proc** | Provides classes and interfaces for executing Flux Balance Analysis processes. |
| **sbrt.kernel.math.algebra.proc** | Provides classes and interfaces for executing algebra-related processes. |
| **sbrt.kernel.math.comb.proc** | Provides classes and interfaces for performing combinatorial analysis-related processes. |
| **sbrt.kernel.math.geom.proc** | Provides classes and interfaces for performing geometry-related processes. |
| **sbrt.kernel.math.graph\_theory.proc** | Provides classes and interfaces for performing graph theoretical processes. |
| **sbrt.kernel.math.stat.proc** | Provides classes and interfaces for performing statistical processes. |
| **sbrt.kernel.utilities.proc** | Provides classes and interfaces for performing utility processes. |
| **sbrt.shell.mng** | Provides classes and interfaces for creating process managers. |

Classes in sbrt.kernel used by sbrt.kernel

****KernelProcess****
  
          This interface is used to represent processes.

Classes in sbrt.kernel used by sbrt.kernel.fba.proc

****KernelProcess****
  
          This interface is used to represent processes.

****MonitoredProcess****
  
          This interface is used to represent monitored
processes.

Classes in sbrt.kernel used by sbrt.kernel.math.algebra.proc

****KernelProcess****
  
          This interface is used to represent processes.

Classes in sbrt.kernel used by sbrt.kernel.math.comb.proc

****KernelProcess****
  
          This interface is used to represent processes.

Classes in sbrt.kernel used by sbrt.kernel.math.geom.proc

****KernelProcess****
  
          This interface is used to represent processes.

****MonitoredProcess****
  
          This interface is used to represent monitored
processes.

Classes in sbrt.kernel used by sbrt.kernel.math.graph\_theory.proc

****KernelProcess****
  
          This interface is used to represent processes.

****MonitoredProcess****
  
          This interface is used to represent monitored
processes.

Classes in sbrt.kernel used by sbrt.kernel.math.stat.proc

****KernelProcess****
  
          This interface is used to represent processes.

****MonitoredProcess****
  
          This interface is used to represent monitored
processes.

Classes in sbrt.kernel used by sbrt.kernel.utilities.proc

****KernelProcess****
  
          This interface is used to represent processes.

****MonitoredProcess****
  
          This interface is used to represent monitored
processes.

Classes in sbrt.kernel used by sbrt.shell.mng

****KernelProcess****
  
          This interface is used to represent processes.

****MonitoredProcess****
  
          This interface is used to represent monitored
processes.

---


|  |  |  |
| --- | --- | --- |
| **Overview** \| **Package** \| Class \| **Use** \| **Tree** \| **Deprecated** \| **Index** \| **Help** | |  |
| PREV   NEXT | **FRAMES**    **NO FRAMES**     **All Classes** |  |


---
